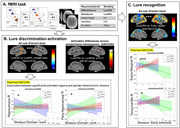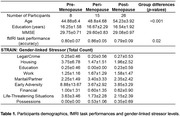# Task fMRI recognition activation at midlife associated with gender‐influenced chronic stressor exposure in women at risk of Alzheimer's disease

**DOI:** 10.1002/alz70856_101652

**Published:** 2025-12-25

**Authors:** Xiaowei Zhuang

**Affiliations:** ^1^ Cleveland Clinic Lou Ruvo Center for Brain Health, Las Vegas, NV, USA; University of Nevada Las Vegas, Las Vegas, NV, USA

## Abstract

**Background:**

Sex‐specific neural activities during memory tasks have been documented across the Alzheimer's disease (AD) continuum^1,2^. Sex and gender further shape stress‐related biological processes regulated by estrogen^3^. Chronic stress in turn impacts brain structure and function differentially by sex, particularly in AD‐vulnerable regions^4^. However, the interaction between gender‐linked stressors, estrogen, and memory‐related neural activities remain under‐explored in women at risk for AD.

**Method:**

We recruited 49 middle‐aged women with a family history of AD (Table 1). Gender‐linked chronic stressor levels were quantified using the STRess and Adversity INventory (STRAIN)^5^, and menopausal stage was determined using Stages of Reproductive Aging Workshop (STRAW) criteria^6^. Functional MRI (fMRI) data during a modified mnemonic similarity task (MST^7,8^) were acquired on a 3‐Tesla Siemens Skyra scanner (TR=1.485s, 2mm isotropic, 524 time‐frames x 3 runs). Everyday objects were first presented in the encoding phase, followed by recognition of identical (targets), similar (lures) and new (foils) objects (Figure 1A). Neural activation patterns were analyzed for Lure recognition (i.e., Lures vs. Foils) and Lure discrimination contrasts [i.e., lures correctly identified as similar (LureCR) vs. falsely identified as same objects (LureFA)]. Post‐hoc ANCOVAs were performed to evaluate whether menopause stage and gender‐linked stressors interact to predict neural activity in contrast‐activated regions.

**Result:**

Perimenopausal women showed the highest recognition accuracies during fMRI task (Table 1). Significant brain activations in the prefrontal, medial temporal and parietal regions were observed for both contrasts (Figure 1). Further, lure discrimination activation was significantly related to task performance. Post‐hoc ANCOVA revealed that, for postmenopausal women, more lifetime chronic stressors occurring early in life and those involving work were related to greater neural activity in the temporal region, whereas pre/perimenopausal women demonstrated reduced or opposite associations (Figure 1).

**Conclusion:**

These findings suggest menopausal status may moderate the association between gender‐linked stressor exposure and temporal lobe activation during a challenging memory task. Future investigations will examine continuous estrogen measures to elucidate the mechanistic basis of this interaction. This research could advance our understanding of how sex‐specific neuroendocrine factors may influence stress‐related cognitive vulnerability in women at high AD risks.